# “You get out of the house, you talk to each other, you laugh…And that’s fantastic” – a qualitative study about older people’s perceptions of social prescribing in mainland Portugal

**DOI:** 10.1186/s12913-024-11086-w

**Published:** 2024-05-20

**Authors:** Andreia Costa, Joana Henriques, Violeta Alarcão, Teresa Madeira, Ana Virgolino, Marie J. Polley, Adriana Henriques, Rodrigo Feteira-Santos, Miguel Arriaga, Paulo Nogueira

**Affiliations:** 1 Nursing Research, Innovation and Development Centre of Lisbon (CIDNUR), Nursing School of Lisbon, Avenida Prof Egas Moniz, Lisbon, Lisboa 1600 - 190 Portugal; 2 Instituto de Saúde Ambiental (ISAMB), Faculdade de Medicina, Universidade de Lisboa, Av. Prof. Egas Moniz, Ed. Egas Moniz, Piso 0, Ala C, Lisboa, 1649-028 Portugal; 3 Laboratório para a Sustentabilidade do Uso da Terra e dos Serviços dos Ecossistemas – TERRA, Av. Prof. Egas Moniz, Ed. Egas Moniz, Piso 0, Ala C, Lisboa, 1649-028 Portugal; 4 Católica Research Centre for Psychological-Family and Social Wellbeing (CRC-W), Faculdade de Ciências Humanas, Universidade Católica Portuguesa, Lisboa, 1649-023 Portugal; 5 Escola Nacional de Saúde Pública, ENSP, Centro de Investigação em Saúde Pública, CISP, Comprehensive Health Research Center, CHRC, Universidade NOVA de Lisboa, Avenida Padre Cruz, Lisboa, 1600-560 Portugal; 6 Centro de Investigação e Estudos de Sociologia (CIES-Iscte), Instituto Universitário de Lisboa (Iscte), Lisboa, 1649-026 Portugal; 7 Laboratório de Nutrição, Faculdade de Medicina, Universidade de Lisboa, Avenida Professor Egas Moniz, Lisboa, 1649-028 Portugal; 8 Research and Development, Meaningful Measures Ltd, Bristol, UK

**Keywords:** Focus groups, Qualitative research, Community participation, Older adults, Active and healthy aging

## Abstract

**Background:**

Social prescribing (SP) is a non-clinical approach, most commonly based in healthcare units, that aims to address non-medical health-related social needs by connecting individuals with community-based services. This qualitative study explores the perception of Portuguese older adults regarding the benefits of SP and their willingness to participate in SP initiatives.

**Methods:**

Three face-to-face focus group sessions were conducted with 23 participants in different cities in Portugal. Open and semi-open questions were used to guide the discussions and thematic analysis was used to analyze the data.

**Results:**

The participants recognized the potential benefits of SP for older adults, including diversifying leisure activities, improving mental health, and complementing existing support systems. They highlighted the need for external support, usually in the form of link workers, to facilitate personalized referrals and consider individual characteristics and preferences. While some participants expressed reluctance to engage in SP due to their existing busy schedules and a perceived sense of imposition, others showed openness to having new experiences and recognized the potential value of SP in promoting activity. Barriers to participation, including resistance to change, mobility issues, and family responsibilities, were identified.

**Conclusions:**

The study emphasizes the importance of a person-centered and co-designed approach to SP, involving older adults in the planning and implementation of interventions. The findings provide valuable insights for the development of SP programs tailored to the unique needs and aspirations of older adults in Portugal, ultimately promoting active and healthy aging. Future research should consider the perspectives of family doctors and include a broader representation of older adults from diverse geographic areas.

**Supplementary Information:**

The online version contains supplementary material available at 10.1186/s12913-024-11086-w.

## Background

Social prescribing (SP) is an approach that enables primary care health professionals (mainly family doctors) to refer patients to a range of local non-clinical services when they face socioeconomic and psychosocial issues that affect their health [1, 2]. These services are typically provided by voluntary and community organizations and offer a wide range of activities (e.g., volunteering, gardening, cooking), serving as a way to improve individuals’ well-being and reduce pressure on health services [1, 2]. Although SP models have been evolving, they traditionally include three key components: the healthcare professional who makes the initial referral, the community organizations that receive the referral, and the link worker who mediates the person’s pathway between them. This figure works closely with the referred person to understand what matters to them, which areas of their life they would like to improve, and what goals they would like to set to improve their health and well-being. The link worker has a motivational and supportive role, but each person is encouraged to take an active part in co-producing the activity plan in order to empower and promote greater autonomy in the management of one’s health [3].

With the unprecedented aging of the population [4], as well as older adults’ elevated risk of developing non-communicable diseases [5], it is imperative to guarantee the best possible conditions to age with quality of life [6], while maintaining sustainable health systems [7]. Even more so when considering the significant impact the COVID-19 pandemic has had on this age group (e.g., increased social isolation), which highlighted the need for effective support systems [8, 9]. Older adults have been shown to benefit from SP interventions [10, 11], but such a heterogeneous group has very unique aging realities (e.g., bereavement, cognitive deficits), so it is expected that different kinds of referrals will be necessary to meet their diverse needs [12]. Generic, ‘one size fits all’ interventions are often less effective as they fail to generate positive outcomes for everyone [13]. That is why it is vital to recognize, evaluate, and cater to individuals’ situations and interests when designing SP services to ensure their adequacy in supporting them [14].

Integrating person-centered principles into the design of SP initiatives is one way to achieve this [15, 16]. By acknowledging and prioritizing individuals’ perspectives, needs, and preferences from the outset, SP interventions can be more effective [16]. Studies have shown that adopting a co-design and co-production approach in SP allows individuals to actively contribute, feel heard, and increase their self-confidence, sense of control, and ownership [17]. Thus, employing this strategy is key to ensuring the sustainability of the intervention, as well as enhancing people’s engagement and utilization [17]. In addition, obtaining consistent feedback from individuals at the beginning of the process allows for an understanding of whether their expectations are being met, identifies anticipated benefits and drawbacks, and reveals potential limitations and barriers they may face when adhering to SP [18]. Such valuable information leads to the development of more engaging, inclusive, and robust SP schemes [17, 19].

Having originated in the United Kingdom, SP has rapidly spread over the world and is currently being developed in 17 countries [20]. One of them is Portugal, where SP is being implemented in some healthcare units in the capital city, Lisbon [21]. The country can be characterized by its markedly aging population (23.8% of the population was over 65 years old in 2022), as well as a considerably high prevalence of chronic conditions, social isolation, loneliness, and mental ill health in older people (previous to the pandemic and exacerbated during this time) [22–25]. Yet, despite this scenario, SP is still not being implemented at a national level. We expect that this approach could help combat the high levels of social isolation found in Portuguese older adults [26], but to the authors’ knowledge, there are currently no available studies exploring this population’s receptivity to SP.

While there is a variety of qualitative SP studies available internationally (e.g [11]), a critical gap in the literature remains. Health professionals and community services are often involved in the early stages of the planning and development process, while the perspectives of the individuals who will participate in SP initiatives are not adequately considered [27]. Only a limited number of studies include service users in the design and planning of the interventions, with the majority focusing on the evaluation phase [28]. This lack of consultation with patients/users has been identified as a limitation in some studies and calls for additional research in this area [29, 30]. For that reason, this study aimed to understand the perception of Portuguese older adults regarding SP’s role in promoting active and healthy aging, as well as exploring their willingness to participate in SP initiatives. By addressing this research gap, the findings of this study aim to contribute to the development of subsequent person-centered SP initiatives tailored to the unique needs, expectations, and preferences of older adults, ultimately promoting their health, well-being, and active aging.

## Methods

### Study design, setting, and sample

This exploratory qualitative study employed semi-structured face-to-face focus group sessions, conducted by an external moderator (social psychologist) who was specifically hired for this role due to her considerable experience. The sessions lasted approximately 90 min each and were conducted in three Portuguese cities: Porto, in the north; Lisbon, in the center; and Lagos, in the south of Portugal. The sessions took place between October 10th and October 30th 2022. The selection of cities was based on their diverse characteristics: Porto and Lisbon represent large metropolitan areas, while Lagos is situated in a municipality with a relatively aging population. This geographical diversity aimed to capture a range of perspectives from older adults in different settings.

Participants were recruited using a non-probabilistic convenience sampling technique from a group of people who had consented to participate in this qualitative data collection in a previous study. The inclusion criteria were that participants should be over 65 years old, able to provide consent, and not have prior knowledge or experience with SP. Given the nature of the recruitment process, the authors tried, as much as possible, to guarantee diversity concerning sociodemographic characteristics (e.g., gender, civil status).

A total of 23 participants attended the focus groups (eight in Porto, eight in Lisbon, and seven in Lagos), of which 14 were female, 13 were married and 18 were retired. The participants’ age ranged from 65 to 90 years old (*M =* 70.9; *SD =* 5.9). Additional sociodemographic details can be found in Table 1.

**Table 1:** Sociodemographic characteristics

	*N*	%
Sex	23	-
Male	9	39.14
Female	14	60.86
Age (*M =* 70.90; *SD =* 5.90)	23	-
Civil status	23	-
Single	2	8.69
Married	12	52.17
Divorced	6	26.08
Widowed	3	13.04
Employment situation	23	-
Still working	3	13.04
Unemployed	1	4.34
Retired	18	78.26
Never worked outside the home	1	4.34

### Measures

The study employed open and semi-open questions to guide the focus group discussions and address the study’s aims. The questions were designed to explore participants’ perspectives on the usefulness of SP for their health and their willingness to participate in SP activities. The question guide was carefully developed for this study (please see Supplementary Material 1) through several rounds of review by the research team to ensure clarity and alignment with the study’s objectives.

### Procedure

Participants were seated around a table in a room and each focus group session commenced with the moderator obtaining written consent from participants. The participants were then provided with an explanation of the focus group methodology, the rules of engagement (e.g., taking turns to speak), and an overview of the study’s aims. Prior to the discussion, definitions of active aging (“the process of optimizing opportunities for health, participation and security in order to enhance quality of life as people age”) [31] and social prescribing (introduced in the first paragraph of the introduction) [2], were provided to ensure a common understanding among participants.

### Data analysis

The focus group sessions were audio-recorded and transcribed verbatim while preserving the anonymity and confidentiality of all participants, by omitting any identifying information. The data was analyzed manually according to the principles of thematic analysis [32]. This methodology was adopted for its comprehensiveness, flexibility and usefulness, which allowed for a combination of the inductive and deductive analytical approaches. The main analysis was performed by one of the authors, being then validated by two others. If consensus remained elusive, other team members were brought in for consultation [33].

**Fig. 1:**
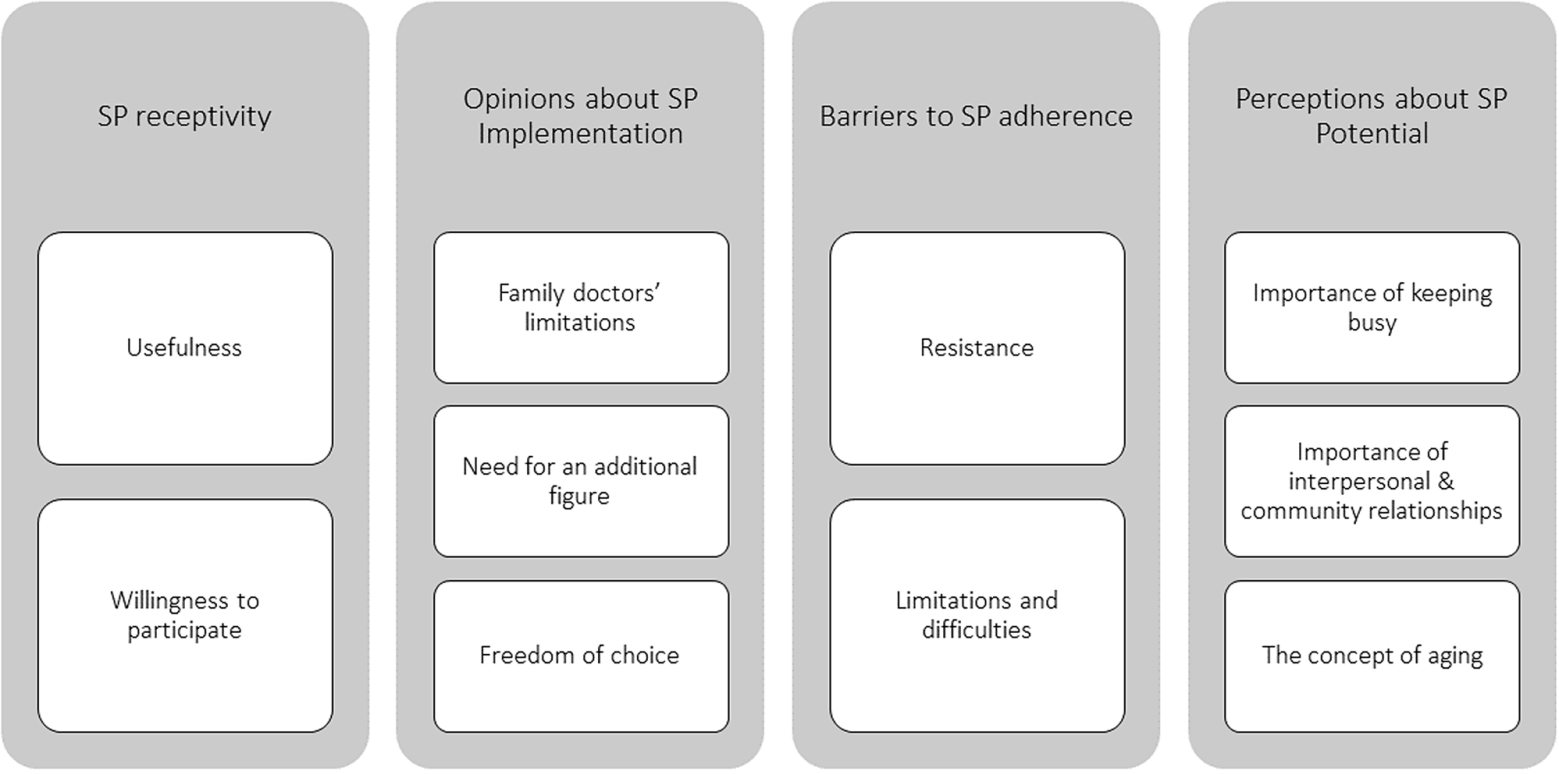
Tree of codes and themes

The categories established *a priori* were related to the research aims (i.e., Usefulness of SP and Openness to SP), but 10 new codes were also created to frame the remaining information (e.g., The concept of aging), resulting in a total of 12 codes. These codes were further analyzed and integrated into four broader themes (see Fig. 1 below). To ensure the consistency of the analysis, a dictionary of codes and themes was developed, providing definitions and illustrative examples (please refer to Supplementary Material 2). The detailed analysis process allowed for a comprehensive understanding of the participants’ perspectives and insights regarding social prescribing and its potential benefits and challenges.

## Results

### SP receptivity

Across all focus group sessions, most participants thought SP seemed useful for older adults, even more so because it allowed them to diversify their interests. People thought it would be beneficial on various levels, spanning from mental health outcomes, to acting like a necessary complement to existing support systems, or even to help activate older people who would otherwise be spending a lot of time indoors, not engaging in stimulating activities.

“From a certain age on, there is sedentarism. Anything that is related to providing points of interest so that people can feel busy, so that they don’t just watch TV or stay cooped up at home (…) It seems like a great solution (social prescribing)” [Participant 1, Porto FG].

Nonetheless, participants appeared to be quite autonomous and resourceful in finding activities and hobbies to be engaged in, recognizing the importance of staying active by themselves and not needing the help of a professional. Consequently, many showed reluctance to the idea of partaking in SP schemes because they were already busy. As they were already engaged in other activities, they seemed to prioritize those over new SP possibilities, and to consider it would be like an imposition, which would have a negative effect.

“Let’s just say that I, like many people here, make my own social prescribing (…) I devote myself to developing my own culture, by reading, studying…” [Participant 4, Lagos].

However, another share of participants seemed open to trying SP to experience new things and to have an extra incentive to be more active. It is noteworthy to mention that even the participants who were not available to try SP at the time, seemed to be willing to do so in the future.

“I think outdoor activities are fundamental (…) volunteering and traveling is important too. Then there are a million things you can come up with [for social prescribing], like organized game rooms with billiards, table football…” [Participant 8, Lisbon FG].

## Opinions about SP implementation

Following the definition provided for SP, which places substantial emphasis on family doctors, participants were adamant in pointing out that they usually have very little time and a lot of patients to see, which in their view made them less suitable to support the SP process. These time constraints seem to lead to a lack of opportunity to focus on non-clinical aspects and might make it more difficult for doctors to see their patients as a whole.

“The other family doctor we had, we didn’t just talk about health, we talked about music. And it’s important to be able to talk about these things, because that’s also part of our health. Now the younger doctors have to see I don’t know how many patients and there’s no time to get to know people” [Participant 7, Porto FG].

For this reason, participants landed on the idea of needing an external figure to support the SP pathway to properly provide benefits. This would be someone who can refer the person to community resources more effectively, because of their ability to get to know the person, along with their characteristics. In fact, it became evident in all focus groups that participants thought that the SP referral should not be generic, but instead take into account how people’s interests, needs, and limitations differ. For that, the professional must consider that and act accordingly, respecting people’s preferences.

“Everyone would have to be guided, according to their temperament and their personality, to suitable activities. And that’s where individual guidance would make sense” [Participant 3, Lisbon FG].”

Following what was described above, participants thought SP should be grounded on people’s free will and ability to choose when and how to be involved, even if that choice meant doing nothing at all. Related to this self-determination idea, was the notion that a social prescription should take on the form of guidance to allow people to be a part of that process, rather than being directive.

“A clinician would tell us what to do, but I don’t agree with that part [moderator asks how it would make sense] they could present three or four options: ‘I think this would suit you’ [moderator asks who would choose] I would. I’d try it out - either it works or it doesn’t. If it doesn’t work, I’ll apologize, say it isn’t for me, and leave” [Participant 7, Lagos FG].

### Barriers to SP adherence

Participants were of the opinion that Portuguese people are resistant to change when faced with new opportunities and that it would prevent them from fully enjoying SP. They believe people show little interest in getting involved and are unwilling to accept alternative activities available in the community, even when they are free. In contrast, participants in the Lagos region shared that immigrants seem more eager to attend local events and activities, possibly due to cultural differences and being more open-minded.

“In relation to social prescribing, I think that people are not willing to accept it (…) people are not very open” [Participant 6, Lagos FG].

In terms of personal limitations and difficulties that might prevent them from engaging in SP, participants mentioned two types of family duties. On the one hand, some were informal caregivers to their relatives. On the other hand, some participants were supporting their children and grandchildren, feeling responsible for helping them financially and by taking care of them after school, which reduced their free time.

“Also the circumstances of life, they lead me to feel that I am (at) the center, the one responsible for the family. I have all this load and I think, I have no free time” [Participant 7, Lisbon FG].

Going beyond familial issues, participants alluded to physical (mobility), health limitations and the lack of adapted infrastructure for older people, related for example to the unavailability of suitable parking spaces or public transportation. The final element that participants brought up as a potential hindrance to the SP process was related to communication. Participants felt it was important to have a functional and timely way of informing people about when and where activities will take place so that they can attend.

“Most retirees retrieve their pensions at CTT (post office). We have to establish partnerships with them to display the information there, so that people know that this week, on such day, between so and so, they can go to such a place and take advantage of things, for example, music and dancing” [Participant 8, Porto FG].

### Perceptions about SP’s potential

The final theme combines ideas about the potential that SP could have on central aspects, shared by the participants, related to positive aging. It was unanimously acknowledged that staying active, having a purpose, and engaging in new experiences led to an enriched life and a positive impact on participants’ well-being. As such, one of the participants in Lagos talked about their experience at the senior university, another in Porto shared the importance they attributed to choir activities, and someone in Lisbon recalled their experience in a community vineyard.

“There isn’t a subject I don’t take [at the senior university], I take part in everything. And it’s a good thing I joined, otherwise I wouldn’t leave the house. Because on the days when I don’t go there, I don’t leave the house” [Participant 3, Lagos FG].

Participants in all locations commented on the current lack of interpersonal and community relationships experienced in modern society, especially in cities, and how that could be countered by SP, particularly highlighting community socialization to reduce social isolation.

“I think it’s very important to work on the neighborhood aspect. I live in a council district with a huge amount of people, but I can’t do anything with the community there” [Participant 7, Porto FG].

Finally, participants in Lagos and Lisbon called forth a discussion about the current concept of aging in light of SP, defending that labeling people as “elderly” after they turn 65 does not seem suitable any longer given how differently people are aging. Simultaneously, they resisted the idea that older people have less value just because of their age, suggesting that they are still interested in life and have a lot to learn, which can be assisted by SP.

“We’re already at an age… but we can still develop, if only intellectually. I think that kind of (mentality) would be good…” [Participant 2, Lisbon FG].

## Discussion

Social Prescribing (SP) is recognized as a non-clinical alternative that can improve the health and well-being of individuals in a holistic manner [1]. This is notable for older adults who face multiple challenges, such as social isolation and cognitive decline which can be addressed through SP interventions [34]. However, many SP initiatives often overlook the perspectives of service users until they are already engaged in the process, missing the opportunity to include their feedback from the outset [17]. Hence, this qualitative study aimed to explore and understand the perceptions of Portuguese older adults regarding the role of SP in promoting active and healthy aging and their willingness to participate in such programs.

The data analysis revealed four themes. The first theme (*SP Receptivity*) evidenced that most participants recognized the potential benefits of SP for older adults, including the ability to broaden leisure options, maintain an active lifestyle, and improve mental health. These findings align with a previous study conducted in Portugal that demonstrated that increased and diversified social and cultural participation among older adults was associated with positive outcomes, such as better self-rated health [35]. In contrast, most participants were already leading quite active lives with a variety of activities and hobbies, which was associated with a sense of independence. Perhaps because of that, they expressed some degree of reluctance to engage in SP. While some participants were open to it, many seemed to associate it with potential disruptions to their existing routines and perceived it as an imposition on their free time. This was a surprising result that is not likely to illustrate older adults’ reality in Portugal, where studies show low activity levels, increased physical disability, and a decline in quality of life during older age [36–38]. This can perhaps be explained by the sampling method chosen, which resulted in a particularly active and autonomous sample with a busy schedule. Nevertheless, it is relevant to mention that even though participants were reluctant about trying SP activities, most believed it would be useful to them in the future when they had more free time and/or for other older adults they knew who were currently not as actively engaged as they were. This finding, in turn, could be more in line with the high rates of social isolation affecting older adults in the country [26].

Through the second theme (*Opinions about SP implementation*) participants expressed concerns about the time constraints faced by family doctors, who are often responsible for referring individuals to SP programs and who might not be able to fully unlock SP’s benefits. Participants believed that doctors may not have enough time to address non-medical aspects due to their heavy workload, leading to a fragmented approach to care. This is in line with what the authors found in a previous study (10.1016/j.pmedr.2024.102652) where a significant proportion of older adults in Portugal claimed to not discuss non-clinical issues during their appointments with health professionals, potentially highlighting the need for additional support. Additionally, there may be indications that one of the challenges family doctors face in relation to SP is remembering to refer patients when the pathway is already in place [39], which could impede their full involvement. Participants emphasized the importance of having an external dedicated figure (usually known as a link worker or social prescriber) who could provide personalized support and understand their specific needs, preferences, and limitations. It is interesting to note that even without learning about the link worker role, which is not included in the SP definition provided, participants were able to glean the importance of such a key element. Much like the literature suggests, interventions must take into account older adults’ needs and preferences to make them feel safe and supported [34]. The participants’ recognition of the importance of person-centered interventions aligns with the existing literature, which highlights the positive impact of tailored approaches on quality of life and health behavior outcomes [15, 16].

Freedom to choose how and when to participate were highly valued by the participants, even if that meant not doing any activities. For instance, a recent study [40] shows that some people do not want to participate in group activities, as was also observed in our participants’ answers. As suggested by that study, it is important to respect people’s wishes (or lack thereof) and consider personal characteristics to find an accommodating fit that makes them feel comfortable [40]. Some participants also expressed a preference for a guiding rather than directive approach, indicating their desire to be actively involved in the decision-making process. Once again it seems that participants were able to land on a core principle of SP, referencing the shared decision-making and autonomy necessary to make them feel empowered and engaged (i.e., “having a voice”) in the SP process [13]. Besides making them feel that they are part of the intervention, it is expected that engaging older adults will improve their experiences and outcomes [41]. On top of the benefits already described, relying more on the link worker rather than the family doctor could circumvent potential access inequities to SP caused by the fact that over 300 thousand people in Portugal were not assigned one – this being especially pronounced in the Lisbon and Algarve areas, where two of the focus groups took place [42].

The third theme (*Barriers to SP Adherence*) included the participants’ perceptions that Portuguese people are resistant to change and have limited interest in unconventional activity options, especially when compared to some of the European immigrants who live in the country and seem more willing to participate. Cultural differences, as well as a need to get settled and feel integrated, may play a role in influencing attitudes toward participation in SP activities and further exploration of these differences could provide valuable insights [24]. Mobility issues, lack of infrastructure, as well as family duties, such as informal caretaking or supporting children and grandchildren, were also identified as obstacles to engaging in SP activities. These identified barriers are consistent with existing literature that highlights the impact of poor health and caregiving responsibilities on older adults’ participation in leisure activities [43–45].

The fourth and final theme (Perceptions about *SP’s Potential*) emphasized the importance of staying active, having a sense of purpose, and maintaining interpersonal relationships and community ties for positive aging experiences. Staying active and having a purpose was believed to have a positive impact on older adults’ well-being and quality of life. Much like our participants’ perceptions, the literature indicates that social engagement and participation have a protective role in cognition and are associated with a higher level of life satisfaction and enhanced well-being [46]. They also highlighted the importance of interpersonal relationships and community ties, sharing how SP is needed to reinforce that. As it is, social support and social integration have been associated with well-being, prevention of chronic conditions, and improved quality of life [46] and have been shown to predict active and successful aging [35]. On the contrary, low neighbourhood cohesion – which was reported by a few participants as well – is associated with negative outcomes (e.g., lower odds of recovery) [46]. Lastly, participants shared that they thought it was reductive to label people as “elderly”. They believed that category should not be used based solely on people’s biological age, since that may not be very indicative of their reality, and that it had a negative connotation related to worthlessness. Other authors [47] have pointed out the need to think more broadly about how to define the “older adults” age group, by contemplating other aspects such as their life expectancy and aging experiences.

### Strengths, limitations, and future research

To our knowledge, this is one of the few studies to explore service users’ preferences, opinions, and suggestions before the SP scheme is designed and developed. As such, this study contributes not only to the growing body of literature on SP internationally but even more so in Portugal where implementation efforts are in the early stages. Furthermore, our results provide encouraging indications about the potential that SP activities could have in promoting active aging among older adults in the country.

Like any study, this one is not without its limitations. Most participants lived in two of the largest urban areas in the country (Porto and Lisbon Metropolitan Areas). Future research should try to include people from rural areas, where there are arguably fewer resources available, to gain a more comprehensive understanding of SP in different contexts. In addition, it became apparent during the focus groups that the participants led particularly active lives and tried to keep as busy as possible in their older age. This seemed to impact their perceptions about the usefulness of SP for themselves, which resulted in them often referring to SP’s potential for other people they knew (second-hand reporting). Future studies should prioritize individuals who are more isolated to explore SP’s suitability more accurately.

Concurrently, exploring the perspectives of family doctors and community professionals in the same locations could provide valuable insights into their views on SP and help identify potential areas for improvement.

## Conclusions

When implementing SP, many decisive factors should be taken into account, such as making sure that older adults are involved in the design and delivery of the intervention and that their insights are considered, so as to meet their unique interests and needs.

This study attempts to provide a first step into understanding the perception and preferences of Portuguese older adults regarding SP and its potential role in promoting active and healthy aging and enhancing social engagement through a sample of individuals in this target group. Our findings highlight the benefits of SP in broadening leisure options, promoting physical and mental well-being, and addressing social isolation, as well as the importance of adopting a personalized and co-designed approach to incorporate older adults’ perspectives and preferences in the development and implementation of SP programs. However, the study also introduced some potential barriers that Portuguese older adults may come to encounter when engaging in SP, including resistance to change, lack of infrastructure, and familial obligations. These findings emphasize the importance of addressing these barriers and tailoring SP interventions to overcome them, taking into account the cultural context and individual characteristics of older adults.

By embracing a person-centered approach, SP has the potential to improve the well-being and quality of life of older adults, supporting their active and healthy aging journey.

### Electronic supplementary material

Below is the link to the electronic supplementary material.

Supplementary Material 1

Supplementary Material 2

## Data Availability

The datasets and materials used and/or analyzed during the current study are available from the corresponding author upon reasonable request.

## Abbreviations

SP: Social Prescribing
WHO: World Health Organization
