# Supplementary material for: “You get out of the house, you talk to each other, you laugh…And that’s fantastic” – a qualitative study about older people’s perceptions of social prescribing in mainland Portugal
Source: BMC Health Serv Res. 2024 May 20;24:645. doi: 10.1186/s12913-024-11086-w (PMC11106980; doi:10.1186/s12913-024-11086-w)
Supplement: Supplementary file 1 — Supplementary Material 1 [file 12913_2024_11086_MOESM1_ESM.pdf]

Focus Group Discussion Guide

1. Objective: To find out the group's perception of Social Prescription in promoting active and healthy ageing.

<u>Framing question: Do you think Social Prescribing would be useful for your health?</u>

Guiding questions:

a) Have you ever heard of Social Prescribing?

b) Has Social Prescribing ever been offered/presented to anyone? If so, could you tell us a bit about that experience?

c) Are there any aspects of your health that you would like to improve?

d) Are there any gains or benefits you would expect to see in your health through Social Prescribing?

e) Are there any barriers or constraints that you personally could experience in relation to Social Prescribing (e.g. adapting the activity to your state of health)?

2. Objective: To find out what role they want to play in Social Prescription (to understand needs and expectations)

<u>Framing question: Would you be willing to participate in Social Prescription activities?</u>

Guiding questions:

a) Has Social Prescription ever been offered/presented to anyone? If so, could you tell us a bit about that experience?

b) How would you react if you were prescribed a Social Prescription activity? Would you do it regularly?

c) What activities would you like to do?

- Physical or intellectual activity (or both...)

- Didactic activities? (learning to use new technologies, etc.)

- Recreational activities? (traditional games

- What do you value more: group or individual activities? (or both...)

- Guided or free? (or both...)

- Outdoors (gardening, walking, ...) or indoors?

d) How do you think the Social Prescription process should take place at the Health Center?

e) How do you think the Social Prescription process should take place in the services in your area of residence?

f) What workshops on healthy eating would make sense to include in the Social Prescription?

g) What intellectual activities (e.g. reading group, chess) would you like to see included in the Social Prescription?

h) Do you think that a more active role on the part of the prescriber (health professional) could help with adherence to the Social Prescription?

i) In general, do you think of possible barriers/constraints when you think about Social Prescribing?

j) Would you talk to a health professional about the possibility of being prescribed these social activities?

k) Even without trying it - and in general - would you recommend a friend to join Social Prescription? Why?
