# Supplementary material for: “You get out of the house, you talk to each other, you laugh…And that’s fantastic” – a qualitative study about older people’s perceptions of social prescribing in mainland Portugal
Source: BMC Health Serv Res. 2024 May 20;24:645. doi: 10.1186/s12913-024-11086-w (PMC11106980; doi:10.1186/s12913-024-11086-w)
Supplement: Supplementary file 2 — Supplementary Material 2 [file 12913_2024_11086_MOESM2_ESM.pdf]

Dictionary of Codes and Themes

Includes a short definition for every code and theme used in the qualitative analysis, along with some illustrative quotes. Since the codes make up the themes, each of them is included in their respective place.

A. SP Receptivity – participants' perceptions towards social prescribing, which includes its usefulness (for themselves or others) and their willingness to participate in social prescription activities

A.1 Usefulness "*I... I think it's beneficial for everybody*" Participant 1 [FG Lagos] / "*I think [social] prescribing should be there, yes. There should be initiatives to tell people what to do, when they don't know what to do, when they feel very alone, for example*" Participant 8 [FG Lisbon]

A.2 Willingness to Participate – the extent to which participants would be interested and willing to participate in social prescription activities that are available or become available in their local context

"*I'd go along with Nature and I'm open to anything. Of course, helping others would also be useful*" Participant 1 [FG Porto] / "*In my case, right now, in 15 years' time I don't know, but right now I wouldn't accept anything, because I have a lot to do. Apart from volunteering*" Participant 8 [FG Lisbon]

B. Opinions about SP Implementation – participants shared ideas about the social prescribing implementation process, mentioning key aspects like challenges from the referral person (doctors), the need for an external support figure (link worker) and the importance of providing a personalized referral (user)

B.1 Limitations of family doctors - following on from the definition of social prescription that was given (with emphasis on the figure of the health professional), the participants consider that family doctors face some limitations and that they are not in the best position to refer users

"*Health doctors, who are the people who should give some guidance, have a time limit for seeing a patient. Unfortunately. They have a time limit and with that time limit, they don't have the capacity to see other difficulties that patients have, to do other types of analysis. Because a patient gets there and complains about who and sometimes even forgets the things they have. And it's impossible for a family doctor, who could provide social guidance for the other services that should be available, to provide guidance, but they don't have that capacity either, because then they have a lot of other patients to see*" Participant 3 [FG Lisbon]

B.2 Need for an additional figure – participants referred to the idea of there being a figure (a professional or an entity) that would do the work of counseling and

monitoring in the social prescription; the descriptions seemed, in general, to resemble the concept of link worker

"*Whether it's done by entities linked to the social management of people, social, society, don't do the doctors, who don't have time to see...*" Participant 3 [FG Lisbon] / "*There must be, there must be some specialists in this to try to indicate to the person, what is the best thing that the person can do or may not want to do at all*" Participant 8 [FG Lisbon]

B.3 Freedom of choice – participants believe in the benefit of social prescription with the condition that activities should only be prescribed in a personalized way to the needs and tastes of each person (they should not be generic); they should not be an obligation and the person should not participate against their will, hence the importance of it being something suitable to them and that is interesting/enjoyable

"*Now, it seems to me that it is a basic thing, but the mentality and the posture of people is very important here, because we have to accept and take things in a way... my father taught me and he used to tell me like this, "everything that was forced, was a difficult thing (...) So, this is a bit important, because then there is a certain tiredness of people and a demotivation when the person is forced to have to do it*" Participant 1 [FG Porto] / "*Others know and others don't know what to do and sometimes a word would be enough to guide... It's not saying: "look, go do this, go do that". It's suggesting, suggesting, because the suggestion is more acceptable than directing, which is almost ordering*" Participant 3 [FG Lisbon]

C. Barriers to SP Adherence – internal or external elements that hinder people's access, involvement and/or adherence to leisure time activities from which they could benefit

C.1 Resistance – resistance to participation in activities and, consequently, to the idea of involvement with social prescription schemes; it partly involves cultural issues of open-mindedness

"*There are very few who participate in that. I mean, it's always the same, same people. It's those 15, ten or fifteen volunteers, but it's always the same*" Participant 7 [FG Lagos] / "*We create about fifty thousand problems, because to mistrust anything, it's the first thing we do. Then and there, we are capable of it. No, we are completely negative to change and change is necessary permanently*" Participant 6 [FG Porto]

C.2 Limitations and difficulties – elements that can somehow hinder the adherence and enjoyment of activities in the community, whether for family issues (e.g., informal caregivers), physical health issues, or lack of information

"*I would really like to do, to participate, to do something, because I don't like to be in a closed house, right? Watching TV is just misery and, unfortunately - as I'm taking care of my husband - he's sick, he's blind, another reason why I can't go out more than*

I'd like to and do something I'd also like to, because I really like to walk in the street and do activities precisely" Participant 2 [FG Lagos] / *"With one or another difficulty that I was talking about, which was the case of mobility, I because of the radiotherapy, the chemotherapy, I have a lot of anemia. One difficulty that I find in being able to go to Oeiras or Cascais - or whatever - is then there is no parking, for example. Public transportation is the shame of it, and then there's no parking. Or being a kilometer away, I don't have the strength to go more than 300 or 400 meters (...) With the anemia I have, it really doesn't work. Even if I want to. Because there is no parking. I mean, you think about everything, but in fact you never think about the fact of the elderly"* Participant 6 [FG Lisbon]

D. SP Potential – more indirectly, participants made some connections between the potential of social prescription and the activation of older people, social and community ties, and active aging

D.1 Importance of keeping busy – sharing about the hobbies, activities, and occupations that participants have engaged in and/or still engage in as seniors, and the importance and impact these have on their lives and well-being

"*But when I went to the Senior University I had never played the ukulele, I had never been into tile painting, I had never been into choral singing [general laughter] I had never been into Italian, I had never been into English, none of that, and I do something every year, don't I? I've been walking for ten years with a ukulele, I've been in tile painting, this year wood painting started...*" Participant 7 [FG Lagos] / *"I work in the church. I go to do the Christmas sale. I organize, I work throughout the year. I worked in the church bar for years, I would leave my house at seven in the morning, take the cakes, work until one or two in the afternoon. I clean the tabernacle. I work, I've been doing that for many years. Already retired, yes*" Participant 4 [FG Lisbon]

D.2 Importance of interpersonal and community relationships – highlights the importance and value of social relationships and community proximity, sometimes being addressed through the negative consequences of the lack/absence of these relationships

"*For example, I think there's a lack of, eh, what's called the neighborhoods, because in the neighborhoods, sometimes people live together a lot, help each other or take care of their kids. I mean, there are a lot of social things that can be done, I think that in Lagos there is not much, there is no neighborhood spirit, it's every man for himself*" Participant 4 [FG Lagos] / "*In this aspect what I notice is that when people, and I live near here, in a building where I don't know people, and I have the habit of running into them and saying hello. There are six floors, about 12 neighbors, but there is no relationship*" Participant 1 [FG Porto]

D.3 Concept of aging – the participants shared some reflections on what it means to be an elderly person, making some criticisms of the definition criteria by the World Health Organization and what it implies, as well as speaking about the importance of active aging and the need to intervene in this sense (prevention and promotion)

"*At 65 [years old] we are elderly, right? So if the WHO said that you're not old at 65, but only at 75 it would drastically reduce the number of old people in the world population, wouldn't it? That's it, because today 62 [years old] is not the 60 [years old] it used to be, right? Today the person who is 60 years old is not elderly*" Participant 4 [FG Lagos] / "*When we see... the older people, I'm not saying "old", I'm saying the older people, older. I don't like to say 'old', I like to say older. Old people are the clothes. I like to say older people. It's society that sees 'old people!*'" Participant 8 [FG Lisbon]
